# Covalent organic imine polymer containing benzothiadiazole as a bifunctional material for specific fluorescence detection and removal of Hg^2+^

**DOI:** 10.1039/d5ra09294a

**Published:** 2026-01-12

**Authors:** Yunrui Gao, Hao Chen, Hanxun Zou, Hang Chen

**Affiliations:** a Fujian Provincial Key Laboratory of Ecological Impacts and Treatment Technologies for Emerging Contaminants, Key Laboratory of Ecological Environment and Information Atlas, College of Environmental and Biological Engineering, Putian University Putian Fujian 351100 PR China

## Abstract

As one of the most toxic heavy metals to humans and the environment, achieving simultaneous fluorescence detection and effective removal of Hg^2+^ presents a significant challenge. In this study, we combined the excellent photoelectric properties of benzothiadiazole groups with the porous structure of covalent organic polymers (COPs) to develop a novel covalent organic polymer material (BTD-MPD-COP) with enhanced fluorescence performance. The benzothiadiazole-modified sites in this material achieved the bifunctional application of BTD-MPD-COP for Hg^2+^ fluorescence detection with a detection limit of 35.65 µM and simultaneous removal with a capacity of 475 mg g^−1^. Furthermore, BTD-MPD-COP demonstrated remarkable Hg^2+^ selectivity and can be reused after desorption without significant reduction in its adsorption capacity. This indicates that BTD-MPD-COP possesses excellent dynamic reversibility in the adsorption of Hg^2+^. In summary, the development of functionalized bifunctional covalent organic polymer materials presents an effective strategy for simultaneous removal and detection of toxic heavy metal ions.

## Abstract 2

As one of the most toxic heavy metals to humans and the environment, achieving simultaneous fluorescence detection and effective removal of Hg^2+^ presents a significant challenge.

## Graphical abstract

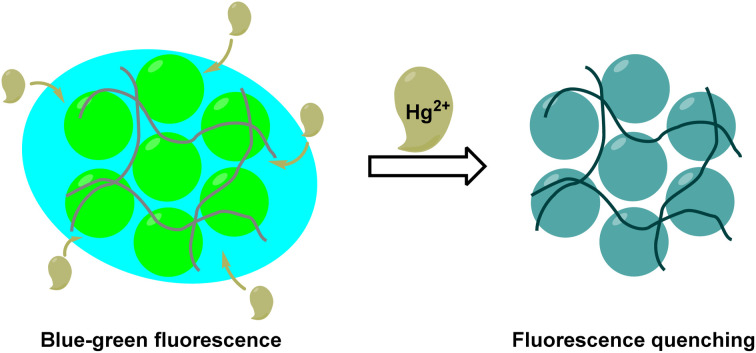

## Introduction

Heavy metal ions are a primary contributor of current environmental pollution, and these pollutants pose a serious threat to human health.^1–6^ Among these pollutants, Hg^2+^ is regarded as one of the most detrimental heavy metal ion contaminants, representing a substantial risk to both human well-being and the ecological environment.^7–12^ Therefore, developing a highly sensitive detection system for Hg^2+^ is of paramount importance, which can help to identify the existence of hazardous sources in a timely manner and eliminate them promptly. Currently, various strategies have been devised to mitigate pollution caused by heavy metal ions, including chemical precipitation,^13,14^ solvent extraction,^15,16^ membrane separation,^17,18^ adsorption^19–22^ and so on. However, each of these treatment techniques presents technical and economic challenges while also being complex and time-consuming in their operational processes. The adsorption method is regarded as one of the most effective ways to remove heavy metals due to its simplicity and low cost. By creating efficient adsorbents specifically designed for this purpose, it is anticipated that this approach will prove effective in eliminating Hg^2+^. Moreover, the optical detection method based on fluorescence or colorimetric changes has become the most convenient and effective method due to their simplicity, rapid response times, and low detection limit, which has become a useful tool for Hg^2+^ detection.^23–26^ Consequently, there is an urgent need for the development of new functional materials that exhibit high adsorption capacity along with excellent selectivity and stability.

Therefore, the concept of designing dual-functional materials capable of both detecting and efficiently removing Hg^2+^ has garnered significant attention. In recent years, various materials such as hydrogels,^27,28^ supramolecular polymers,^29^ metal–organic frameworks (MOFs),^30,31^ and covalent organic frameworks (COFs)^32^ have been employed for the adsorption of Hg^2+^. However, the long reaction time, complex purification process, and poor repeatability associated with these synthetic conditions have largely constrained their potential in practical dual-functional applications. Among these materials, covalent organic polymers (COPs) as a new type of functional material have attracted much attention in the field of materials science due to their unique topological configuration and customizable molecular properties.^33–36^ As a type of porous material connected by robust covalent bonds (such as imine bonds), COPs exhibit great promise in catalysis,^37^ adsorption,^38^ energy storage,^39^ and chemical sensing^33,40^ due to their high specific surface area, adjustable pore size, excellent structural stability, and abundant functional sites. In comparison to COFs, the tight stacking order between layers of COFs usually leads to a strong aggregation-induced quenching (ACQ) effect, resulting in poor fluorescence detection performance.^41^ The disordered structure of COPs can mitigate the ACQ effect. Furthermore, COPs feature a rich conjugated system that enhances electron transport capabilities while exhibiting strong fluorescence characteristics. Consequently, they may represent ideal dual-functional materials for both fluorescence detection and removal of Hg^2+^.

The conventional COPs typically exhibit weak interactions with Hg^2+^, leading to suboptimal performance in the rapid detection and removal of this ion. To develop efficient, dual-functional materials for the swift detection and elimination of Hg^2+^ in aqueous solutions, it is essential to modify COPs by incorporating functional groups that bestow them with these dual capabilities. In this study, we combined the excellent photoelectric properties of the benzothiadiazole group with the unique porous structure inherent to COPs to construct a novel covalent organic polymer known as BTD-MPD-COP. Owing to its rich π-conjugated network, stable porous architecture, and sulfur-functionalized groups, this material demonstrates sensitive fluorescence detection alongside effective removal of Hg^2+^ from aqueous environments.

## Results and discussion

**Scheme 1:**
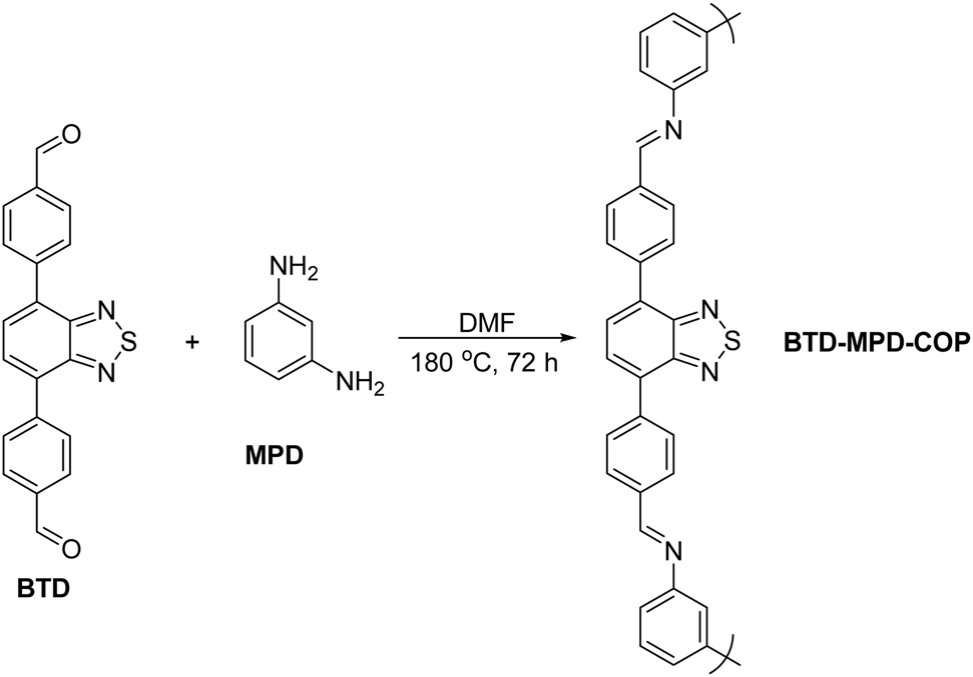
Synthetic routes of BTD-MPD-COP.

The benzothiadiazole-modified COP (BTD-MPD-COP) was synthesized through imine condensation of aldehydes and amines, as illustrated in Scheme 1. The process involved dissolving benzothiadiazole (BTD)-containing monodialdehyde and *m*-phthalimide (MPD) in an appropriate volume of DMF solution. After thorough mixing and ultrasonic treatment for 1 hour, the mixture was transferred to a 25 mL reaction vessel and placed in an oven at 180 °C for 72 hours. Following cooling to room temperature, the crude product underwent three washes with DMF followed by anhydrous ethanol. The final product was vacuum-dried at 90 °C for 24 hours, yielding a approximately 85% reddish-brown powder.

**Fig. 1:**
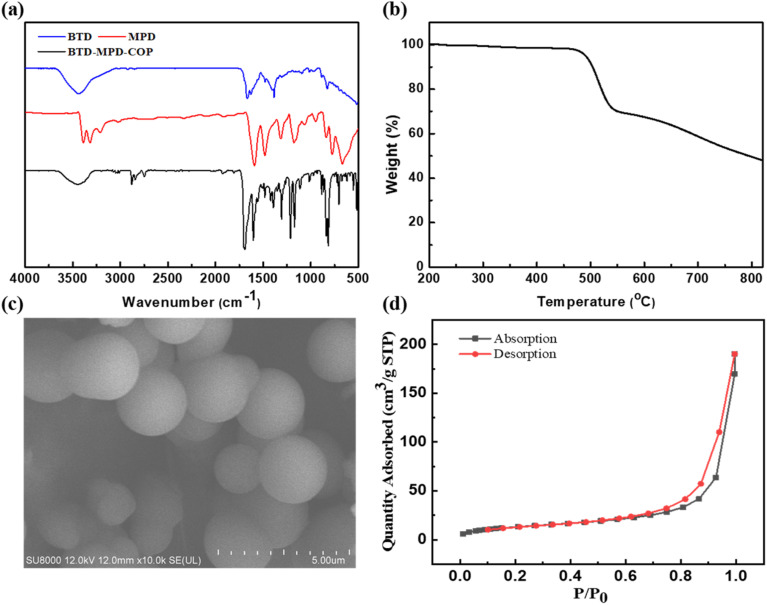
(a) FT-IR spectra of BTD (blue), MPD (red) and BTD-MPD-COP (black) in the wavenumber range 500–4000 cm^−1^. (b) TGA Cures of BTD-MPD-COP under an N_2_ atmosphere. (c) SEM images of BTD-MPD-COP. (d) N_2_ adsorption–desorption isotherms of BTD-MPD-COP.

The Fourier Transform Infrared Spectroscopy (FT-IR) analysis revealed a strong N–H peak at 3000 cm^−1^ corresponding to *m*-phenylenediamine, which gradually diminished in BTD-MPD-COP, indicating complete consumption of the amine component. A new C=N peak emerging at 1623 cm^−1^ confirmed the successful synthesis of imine bonds as well as thiazole-containing organic covalent materials (Fig. 1a). Furthermore, the stability of BTD-MPD-COP was investigated. The thermogravimetric analysis (TGA) was conducted under nitrogen atmosphere to investigate the stability of BTD-MPD-COP which demonstrated that BTD-MPD-COP exhibited no significant weight loss below 450 °C, indicating structural stability up to this temperature. At 800 °C, approximately 50% of the material remained intact, thereby demonstrating the high thermal stability of our covalent organic polymer (Fig. 1b). In order to investigate the surface structural characteristics of the material, the scanning electron microscope (SEM) was employed to observe its morphology (Fig. 1c). BTD-MPD-COP displayed a relatively regular spherical structure with uniformly sized pores alongside multiple pores of varying dimensions, which effectively provided channels for Hg^2+^ adsorption. Nitrogen adsorption–desorption experiments conducted at 77 K revealed isotherms consistent with Type IV N_2_ adsorption profiles—a hallmark characteristic indicative of mesoporous materials.^42^ The calculated specific surface area of BTD-MPD-COP was found to be 47.9 m^2^ g^−1^ with pore diameters measuring approximately 20.5 nm, further substantiating its mesoporous properties (Fig. 1d).

And then the selective fluorescence detection properties of COP materials for heavy metal ions in aqueous solutions were investigated. Among ten common metal ions, only Hg^2+^ significantly quenched the fluorescence signal of BTD-MPD-COP. Fig. S1 and 2a, respectively demonstrated the fluorescence emission spectra and relative luminescence intensity changes of BTD-MPD-COP after metal ion addition. The quenching of the fluorescence intensity may be caused by the interaction between S atoms and Hg^2+^ in the BTD-MPD-COP structure, which hindered the excited proton transfer process.^43^

**Fig. 2:**
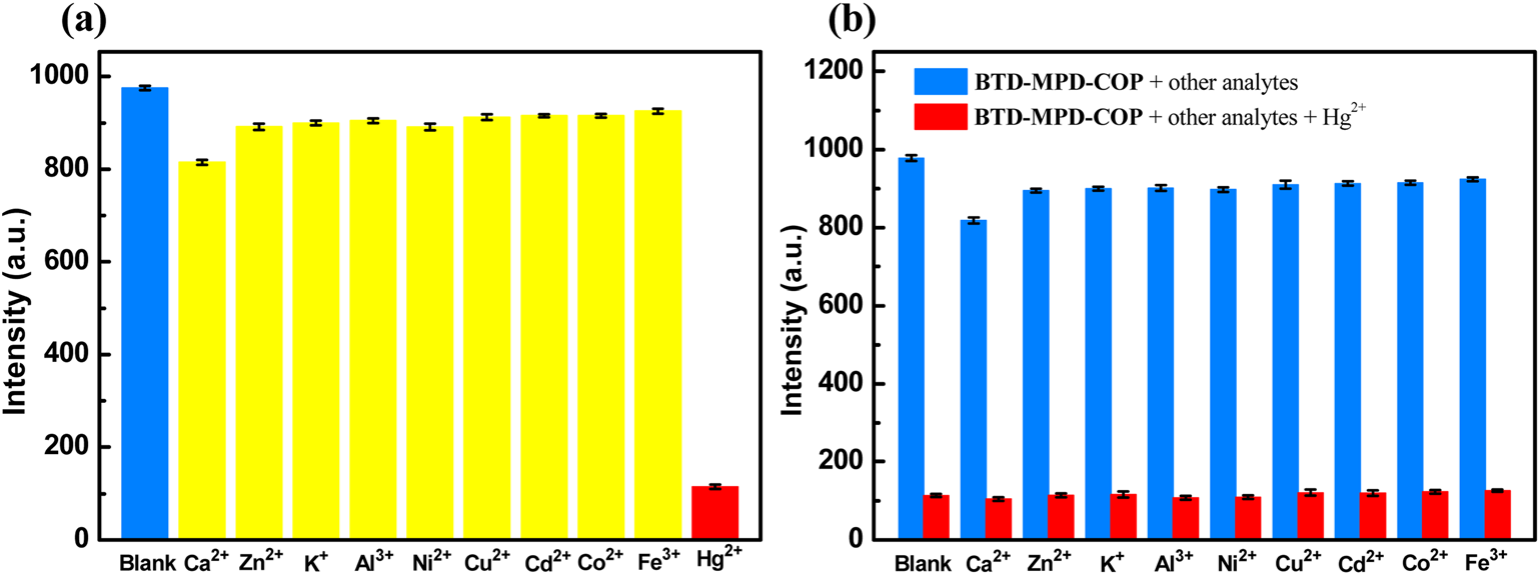
The luminescence intensities of the COPs after adding different metal ions in DMSO solution (1 × 10^−3^ M). (a) BTD-MPD-COP; (b) the luminescence intensities of BTD-MPD-COP after adding other metal ions (10^−3^ M) except Hg^2+^ (black bars) and upon adding Hg^2+^ (10^−3^ M) to the above solutions (red bars);

In order to further investigated the competitive experiment of BTD-MPD-COP for Hg^2+^ detection, the effects of other metal ions on fluorescence intensity changes were tested before and after adding Hg^2+^ (Fig. 2b). The results showed that when competitive metal ions were present, the emission intensity of BTD-MPD-COP solution significantly decreased after Hg^2+^ addition, indicating that these competing ions can not interfere with Hg^2+^ detection. These findings demonstrated that our BTD-MPD-COP exhibits high selectivity for Hg^2+^ detection.

**Fig. 3:**
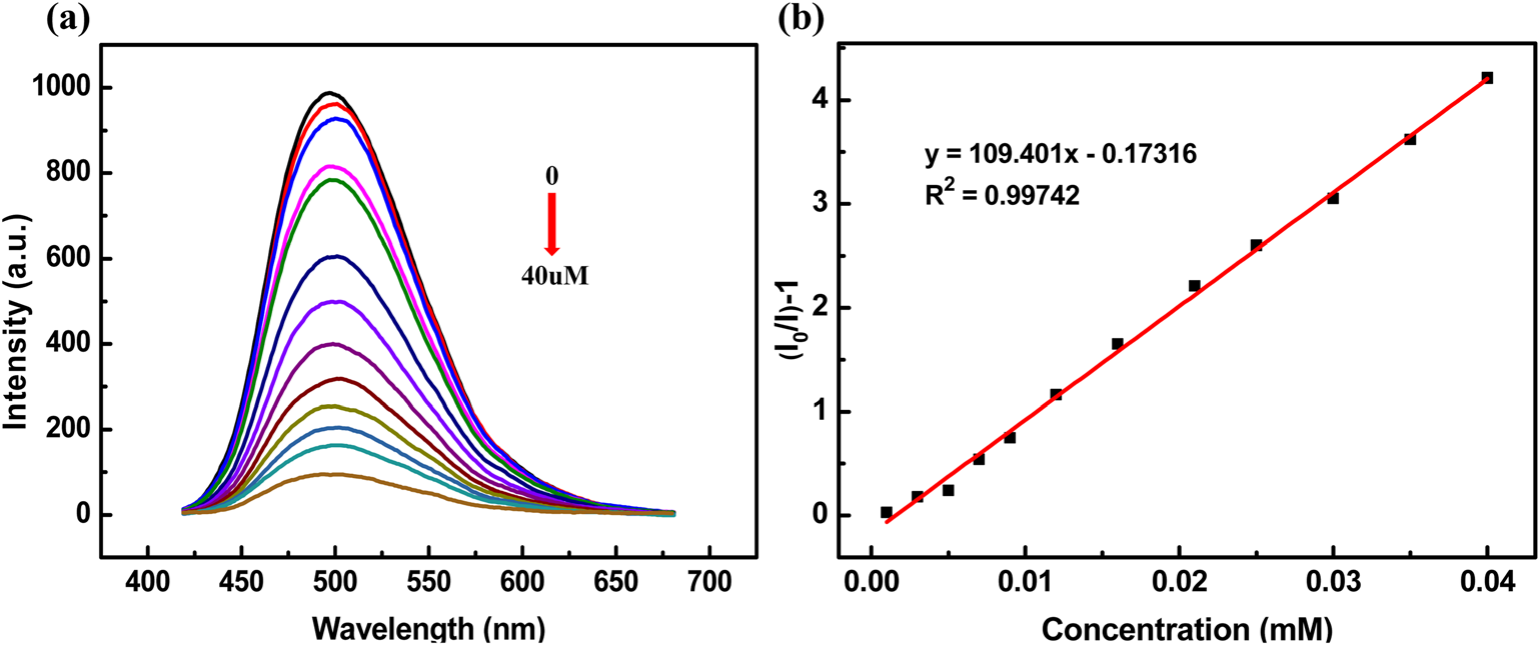
(a) The fluorescence titration of BTD-MPD-COP dispersed in DMSO solution after gradually adding Hg^2+^; (b) Stern–Volmer plots of BTD-MPD-COP for Hg^2+^.

To accurately assess the detection sensitivity of BTD-MPD-COP for Hg^2+^, the real-time fluorescence titration experiments were conducted. As shown in Fig. 3a, the fluorescence intensity of BTD-MPD-COP gradually decreased to 89% through adding 1.0 mM Hg^2+^. Based on the titration results, the quenching coefficient *K*_SV_ was calculated using the Stern–Volmer equation:^44^*I*_0_/*I* = 1 + *K*_SV_ (mM), where *I*_0_ and *I* represent the fluorescence intensity before and after analyte addition. Fig. 3b demonstrated the excellent linear relationship between BTD-MPD-COP and the Stern–Volmer equation in detecting Hg^2+^, with a *K*_SV_ value of 109.401 mM^−1^. Subsequently, the detection limit (LOD) of BTD-MPD-COP for Hg^2+^ was determined as 35.65 µM (Fig. S2), indicating that this fluorescent probe exhibited superior performance in Hg^2+^ detection compared to other studies (Table S1).

In addition, BTD-MPD-COP was left undisturbed with ten different 0.01 mol L^−1^ metal ion aqueous solutions for approximately 24 hours. EDS analysis was performed after drying the materials (Fig. S3). The postadsorption material exhibited a structure composed of multiple spherical aggregates with uneven surfaces. This phenomenon likely arises from the adsorption of mercury or other metal ions on the surface, which occupies available spaces and leads to particle aggregation. Comparative analysis of elemental distribution patterns and layers revealed that metal elements were uniformly and randomly distributed across the material surface. As shown in Table S2, BTD-MPD-COP primarily adsorbed mercury ions, with the mercury content in the material reaching 75.88%, which indicates the presence of mercury adsorption on the surface of the material. Notably, the mercury content was at least ten times higher than that of other metal ions, demonstrating that BTD-MPD-COP exhibits superior selectivity for mercury ions.

**Fig. 4:**
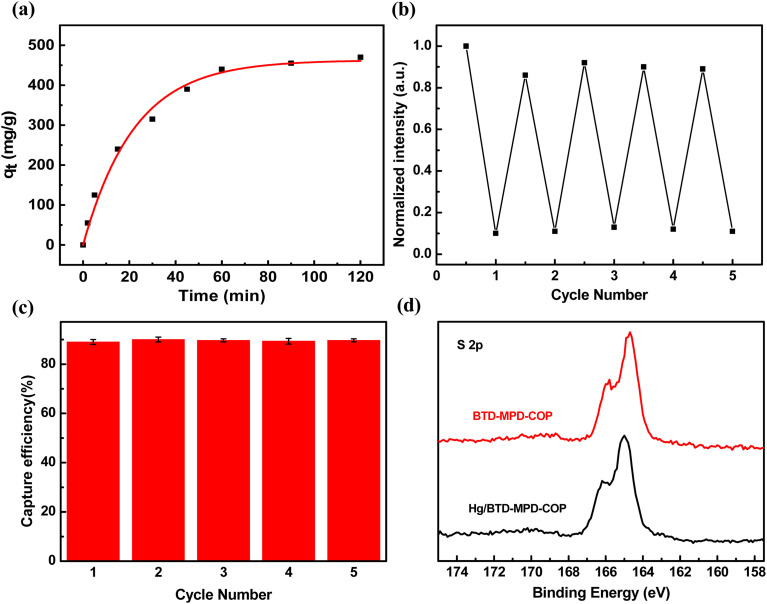
(a) Adsorption kinetic model of BTD-MPD-COP. (b) Recycle use of BTD-MPD-COP for fluorescence detection of Hg^2+^. Upon treatment in aqueous H_2_SO_4_–NaCl solution. (c) Recycle performance of BTD-MPD-COP for facile removal of Hg^2+^ in aqueous solution. Upon treatment in aqueous H_2_SO_4_–NaCl solution. (d) The XPS spectra of S 2p.

As a bifunctional material, BTD-MPD-COP not only demonstrated excellent fluorescence detection performance but also exhibited a high adsorption capacity for Hg^2+^. Therefore, the absorption and removal characteristics regarding Hg^2+^ in aqueous solution were further investigated. Fig. 4a illustrates the adsorption kinetics model of BTD-MPD-COP, the experimental results indicated that in an initial Hg^2+^ concentration of 600 mg L^−1^, the equilibrium adsorption capacity reached approximately 475 mg g^−1^ within about one hour of reaction time and approximately 80% of this capacity was achieved during this period. This is primarily attributed to the uniform pore size and large specific surface area of BTD-MPD-COP material, which creates strong adsorption forces between the material sites and mercury ions. These characteristics correspond to the specific surface area results obtained from BET tests.

Circulation performance is another critical aspect in practical applications. We found that BTD-MPD-COP materials pre-adsorbed with Hg^2+^ can be effectively desorbed using an H_2_SO_4_–NaCl solution, and the treated material was easily regenerable. The regenerated BTD-MPD-COP can be reused for subsequent Hg^2+^ detection and removal processes. Notably, the fluorescence property of the regenerated BTD-MPD-COP was also recovered completely (Fig. 4b). After the adsorption–desorption cycle of Hg^2+^ was repeated five times, the sensitivity and responsiveness of BTD-MPD-COP remain virtually unchanged, and the Hg^2+^ uptake of BTD-MPD-COP almost retained 90% of the original Hg^2+^ capacity (Fig. 4c). These results indicate that BTD-MPD-COP material is chemically stable in the desorption process and has excellent recycling performance.

In order to further confirm the S–Hg interaction in BTD-MPD-COP for selective detection and effective removal of Hg^2+^, X-ray photoelectron spectroscopy (XPS) was employed to analyze the changes before and after BTD-MPD-COP adsorbed Hg^2+^. The binding energies (BE) of S 2p in BTD-MPD-COP and Hg/BTD-MPD-COP were measured at 164.73 eV and 165.02 eV, respectively (Fig. 4d). Compared with BTD-MPD-COP, Hg/BTD-MPD-COP exhibited a positive shift of 0.29 eV for the S binding energy, confirming the interaction between Hg^2+^ and S species where Hg^2+^ withdraws electrons, placing S atoms in an electron-deficient state, which is consistent with the phenomenon commonly observed in other porous materials.

## Conclusions

In summary, the porous luminescent covalent organic imine polymer containing benzothiadiazole (BTD-MPD-COP) was successfully synthesized through a hydrothermal method. This material not only demonstrates selective fluorescence detection but also exhibits high efficiency in the removal of Hg^2+^ ions from aqueous solutions. Our research indicates that the incorporation of imine bonds to enhance long-range π-conjugated structures significantly improves the fluorescence performance of the material, while the S–Hg interaction substantially increases its adsorption capacity for Hg^2+^. Furthermore, BTD-MPD-COP displays a high saturation capacity for Hg^2+^ and excellent cycling stability, allowing for repeated use after desorption without significant loss of adsorption capability, which can prove widely applicable for rapid adsorption and removal of Hg^2+^ in aqueous environments. Indeed, this work presents a universal strategy for developing high-performance bifunctional materials by introducing functional groups to modify COPs.

## Conflicts of interest

The authors declare no conict of interest.

## Supplementary Material

RA-016-D5RA09294A-s001

## Data Availability

The data supporting this article have been included as part of the supplementary information (SI). Supplementary information: the further experimental details of BTD synthesis. See DOI: https://doi.org/10.1039/d5ra09294a.
