# Supplementary material for: Covalent organic imine polymer containing benzothiadiazole as a bifunctional material for specific fluorescence detection and removal of Hg2+
Source: RSC Adv. 2026 Jan 12;16(3):2408–14. doi: 10.1039/d5ra09294a (PMC12793986; doi:10.1039/d5ra09294a)
Supplement: RA-016-D5RA09294A-s001 [file RA-016-D5RA09294A-s001.pdf]

Supplementary Information (SI) for RSC Advances.
This journal is © The Royal Society of Chemistry 2025

Supporting Information

Covalent organic polymer containing benzothiadiazole as bifunctional material for specific fluorescence detection and removal of Hg^{2+}

Yunrui Gao,[a‡] Hao Chen,[a] Hanxun Zou,[a*] Hang Chen,[a*]

a Fujian Provincial Key Laboratory of Ecological Impacts and Treatment Technologies for Emerging Contaminants, Key Laboratory of Ecological Environment and Information Atlas, College of Environmental and Biological Engineering, Putian University, Putian, Fujian 351100, PR China

Table of Contents

1. General Methods……………………………………….....S3

2. Materials and Synthetic Procedures ………………….....S4-S5

3. Supporting Tables and Figures…………………...…...S6-S8

4. References…………………………………………….....S9

1. General Methods

^{1}H NMR and ^{13}C NMR spectra were recorded on a 400 MHz Bruker Biospin avance III spectrometer. Deuterated reagents for characterization were purchased from Sigma-Aldrich Chemical Co. and Cambridge Isotope Laboratories Inc. (purity ≥ 99.9%). The chemical shifts (δ) for ^{1}H NMR spectra, given in ppm, are referenced to the residual proton signal of the deuterated solvent. Mass spectra were recorded on a Bruker IMPACT-II or ThermoScientificLCQ Fleet spectrometer. All other reagents were obtained from commercial sources and were used without further purification, unless indicated otherwise. The functional group analysis of the treated samples was performed using the TENSOR 27 FT-IR spectrometer (FT-IR Spectrometer, FTIR) from Bruker Germany. The process began with spectral calibration using air as the reference, followed by spectral measurements of the treated samples in the range of 8000-350 cm^{-1} with a resolution of 0.25 cm^{-1}. The optical properties were evaluated using the Varian Cary 500 Scan UV-Vis spectrometer in the ultraviolet (UV) region. The compound solution was measured for absorbance with dimethyl sulfoxide as the reference medium. Thermal stability testing of the material was performed using the Waters SDT 650 thermogravimetric analyzer (TGA). Two small crucibles of identical specifications were pre-balanced and filled with a small amount of test material on the left side. Test conditions included: nitrogen atmosphere, heating rate of 10℃/min, temperature range of 30-1000℃, and recording of temperature-programmed thermal curves to monitor mass loss and evaluate thermal performance. The morphology of the COPs was examined using a SU-8010 scanning electron microscope. The materials before and after metal ion adsorption were directly mounted on conductive adhesive tapes, gold-coated via an ion sputter, and observed under an electron microscope at 15 kV acceleration voltage. Clear microscopic images were obtained by adjusting parameters including acceleration voltage, scanning speed, probe current, as well as moving the stage and modifying magnification, focal length, and brightness. The porous properties and specific surface area of COPs were investigated using the ASAP2460 specific surface area and micropore physicochemical adsorption instrument from Macom, Inc. (USA), with nitrogen adsorption-desorption analysis. The procedure involved: first, weighing the BET empty tube and a specified amount of sample; then, degassing the material by heating at 200℃for 8 hours to remove moisture; subsequently, measuring the combined mass of the degassed BET tube and product; finally, inputting the data into the COPs BET analysis template to calculate the specific surface area through nitrogen adsorption-desorption mass changes.

2. Materials and Synthetic Procedures

Synthesis of BTD: 4,4'-(benzothiadiazole-4,7-diyl)dibenzaldehyde (**BTD**) was synthesized according to previous literature.[1]

To a mixture of 4,7-bis(bromomethyl)-2,1-benzothiadiazole (1.0 mmol, 0.28 g), tetraformylphenylboric acid (6 mmol, 0.90 g), cesium carbonate (6 mmol, 1.95 g), and tetrakis(triphenylphosphine)palladium (0.1 mmol, 0.12g), was added 1,4-dioxane (10.0 mL) and H_2O (3.0 mL) under N_2 atmosphere. The reaction was stirred at 85 ℃ for 12 h. After the reaction was cooled to room temperature, H_2O (25 mL) was added, and the mixture was extracted with EA (25 ml × 3). The combined organic layer was washed with saturated aqueous NaCl (50 ml × 3) and dried over anhydrous Na_2SO_4. After removal of solvent under vacuum and further purification performed on column chromatography (silica gel, dichloromethane : methanol = 20 : 1), the title compound was obtained as a green solid (309 mg, 90%). ^{1}H NMR (400 MHz, CDCl3, ppm) δ = 10.13 (s, 2H), 8.17 (d, J = 8.2Hz, 4H), 8.07 (d, J = 8.2Hz, 4H), 7.91 (s, 2H).

Synthesis of BTD-MPD-COP: The synthesis of COP was synthesized through imine condensation of aldehydes and amines. The specific synthesis methods are as follows. **BTD** (0.05 mmol, 0.0167 g) and **MPD** (0.05 mmol, 0.0054 g) were dissolved in dry N,N-dimethylformamide (DMF) (10 mL). And then thorough mixing and ultrasonic treatment for 1 hour, the mixture was transferred to a 25 mL reaction vessel and placed in an 180 °C oven for 72 hours. After cooling to room temperature, the crude product undergoed three washes with DMF and anhydrous ethanol respectively, and dried in a vacuum oven at 90 ℃ for 24 h, yielding a reddish-brown powder.

Hg^{2+} sorption kinetics. Preparing the Hg^{2+} aqueous solutions with gradient standard concentrations, measuring the absorbance at each concentration (2-3 measurements per concentration) and calculating the average values to plot a standard curve of concentration versus absorbance. Placing the **BTD-MPD-COP** and specific mercury concentrations in conical flasks, collecting 3 ml of solution at 0, 2, 5, 15, 30, 45, 60, 90, and 120 minutes, and then measuring the absorbance A_t at specific wavelengths to determine the adsorption kinetics of the samples.

Fluorescence performance study. To evaluate the adsorption capacity of **BTD-MPD-COP** material, the molecular fluorescence spectrometer was used to measure the fluorescence intensity of the samples. The 0.01 mol/L Hg^{2+} solution was prepared with water and ultrasonicated for 30

minutes to ensure complete dissolution. Fluorescence titration was conducted between the Hg^{2+} solution and **BTD-MPD-COP** at specific ratios, and the fluorescence intensity was measured under 365 nm (excitation wavelength) for 3 times measurements per ratio respectively. In fluorescence analysis, the Stern-Vomer equation was used to determine the detection range, and the minimum detection limit (LOD) for mercury ions was calculated using the LOD formula.

Stern-Vomer formula: $I_0/I = 1 + K_{SV}(M)$, LOD calculation formula: LOD = 3 SD / K

Here, I_0 and I represent the fluorescence intensities of the probe before and after pollutant addition, M denotes the concentration of mercury ion pollutant (mmol/L) in the detection, K is the slope of the linear fit of the Stern-Voigt equation, and SD indicates the standard deviation of 10 blank samples.

Selective adsorption of Hg^{2+}. To evaluate the adsorption capacity of **BTD-MPD-COP** material for various metal ions, this experiment compared fluorescence intensities of different ions in material solutions and analyzed elemental distribution using EDS. In fluorescence selectivity experiments, 0.01 mol/L aqueous solutions of different metal ions were prepared. Under conditions where other metal ions were present, appropriate amounts of mercury ion solution were added to measure fluorescence intensity and analyze selectivity. For EDS analysis of adsorption selectivity, each bottle contained 200 uL of ten different 0.01 mol/L metal ion solutions and corresponding sample materials. After 24 hour incubation, the samples were dried and analyzed using EDS to determine elemental distribution patterns.

Circulation performance study. Recyclability serves as a critical parameter for evaluating adsorbent materials. To assess the recyclability of **BTD-MPD-COP**, the desorption solution of H_2SO_4-NaCl with a molar concentration ratio of 1:12.5 was prepared through deionized water. The mercury-loaded **BTD-MPD-COP** was neutralized with H_2SO_4-NaCl solution, and then rinsed with deionized water after ultrasonic treatment for 6 hours. The regenerated material was then recycled for subsequent fluorescence measurements. Finally, the regenerated material was then readsorbed with a 0.01 mol/L Hg^{2+} aqueous solution to compare the removal efficiency of Hg^{2+} before and after desorption.

3. Supporting Figures

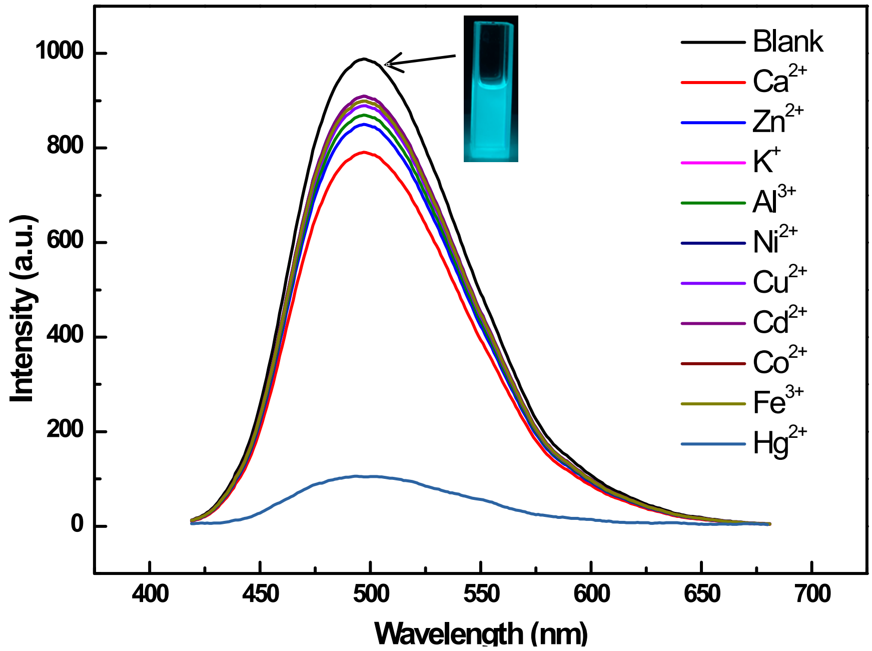

Figure S1. Fluorescence emission spectrum of **BTD-MPD-COP** dispersion with a concentration of 2 mg / 10 ml to detect various metal ions.

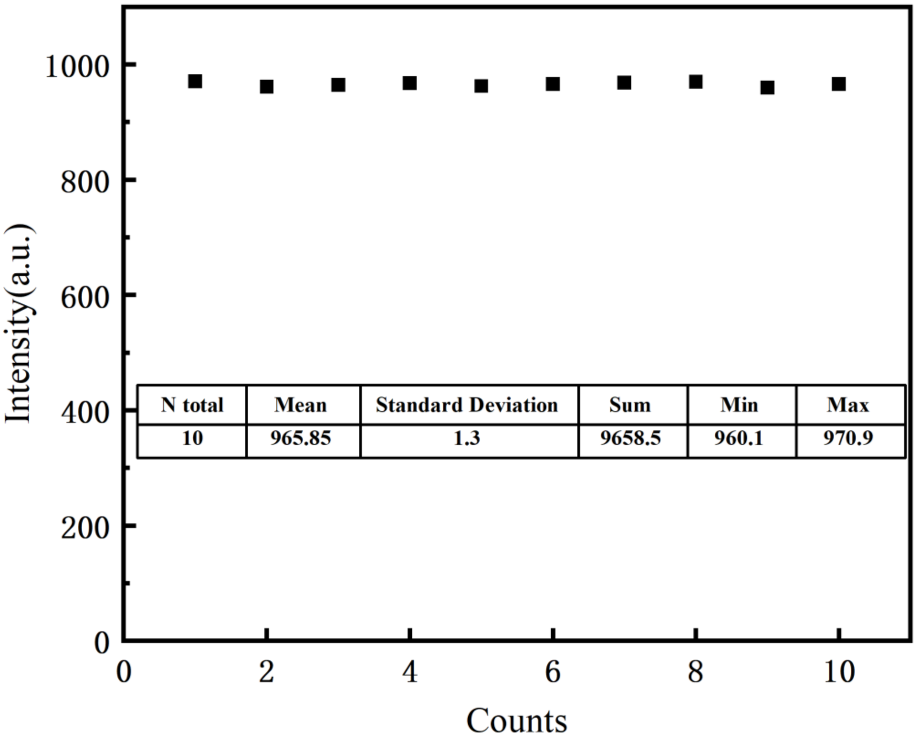

N total	Mean	Standard Deviation	Sum	Min	Max
10	965.85	1.3	9658.5	960.1	970.9

Figure S2. Stability test of **BTD-MPD-COP** fluorescent probe. The corresponding limit of detection (LOD) was determined as 35.65uM using the equation LOD = 3 × S.D. / Ksv, where Ksv represents the slope of the Stern-Volmer plots, and S.D. is the standard deviation for the fluorescence intensity of **BTD-MPD-COP** in the absence of Hg^{2+} .

Table 1 Comparison of the Performance of Hg^{2+} Fluorescent Probes Reported in Different Literature

Material Type	Probe Molecule	Hg^{2+} Detection Limit	Literature
COPs	COP-108-S	1.4 mM	[2]
COPs	TBN-S-SH COP	0.5 nM	[3]
COPs	HTDP-2	0.70 ppm	[4]
COFs	COF-NH_2	15.3 nM	[5]
COFs	COF-TFPB-TS	87 nM	[6]
MOFs	NXS@ZIF-8	1.5 nM	[7]

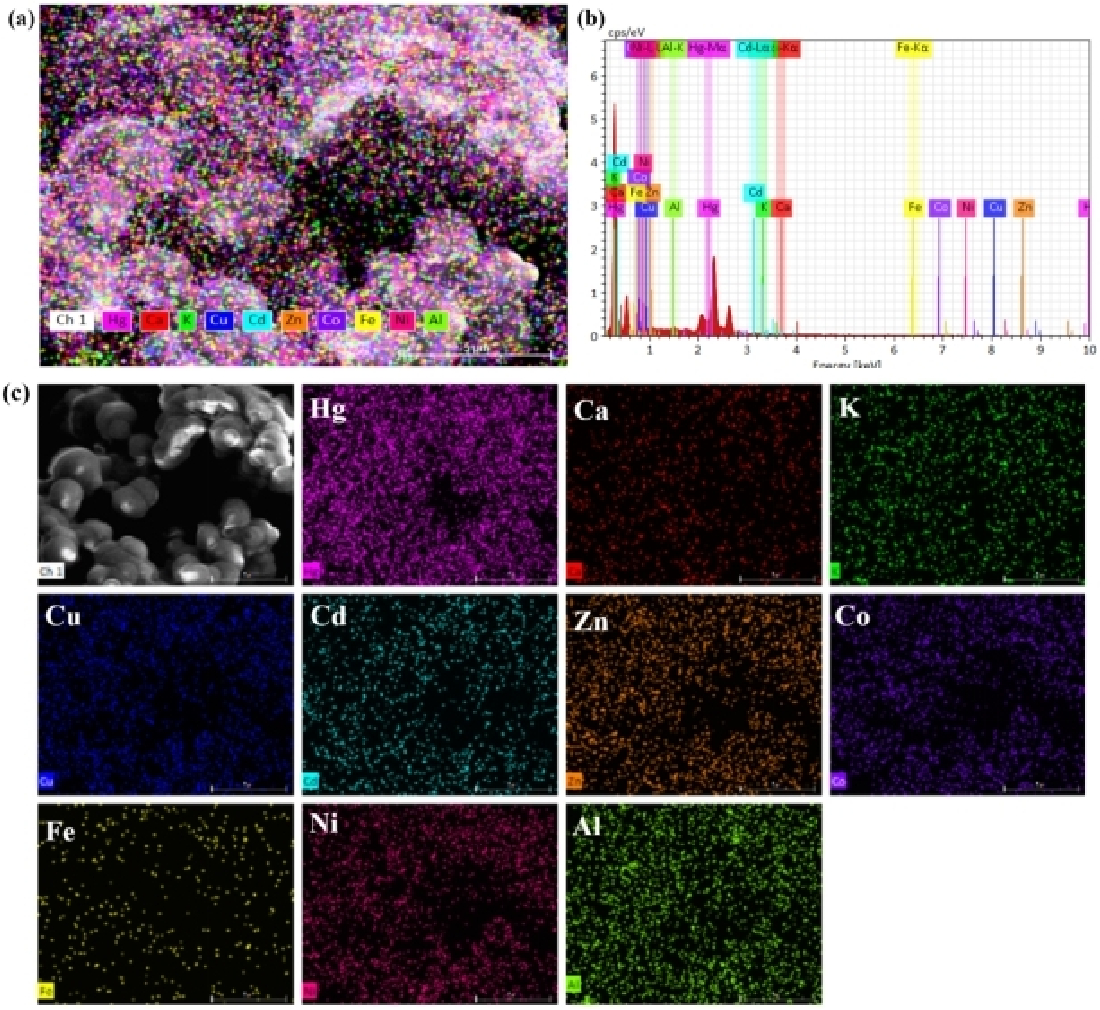

Figure S3. SEM image and EDS elemental distribution of **BTD-MPD-COP** after adsorbing mercury ions. (a) Morphological image after metal ion adsorption; (b) Elemental distribution map; (c) Elemental distribution layer.

Table S2. Elemental Composition of **BTD-MPD-COP** in EDS

Element	Atomic %
Hg	75.88
Ca	5.19
Al	3.24
Zn	4.01
Ni	3.15
Co	1.87
Cd	2.06
Fe	2.53
Cu	2.07

4. References

[1] L. Wang, Q. Xia, M. Hou, C. Yan, Y. Xu, J. Qu and R. Liu, A Photostable Cationic Fluorophore for Long-Term Bioimaging. *J. Mater. Chem. B.,* **2017**,5, 9183-9188.

[2] J. Li, S. Wang, X. Cao, H. Huang and D. Cao, Sulfur-modified porous covalent organic polymers as bifunctional materials for efficient fluorescence detection and fast removal of heavy metal ions. *Mater. Chem. Front.,* **2021**, 5, 3428

[3] Y. Du, H. Shan, Y. Jiang, Z. Zhang, J. Jiao, M. Wang, D. Cai, R. Zhang ang T. Jiao, Bifunctional membrane design based on thiol-functionalized triazine-containing covalent organic polymer for integrated mercury ion removal and monitoring. *J. Membr. Sci.,* **2025**, 733, 124316.

[4] Y. Zhu, C. Ni, C. Zhang, Z. Liu, G. Wu and Y. Qin, Dual-function Covalent Organic Polymers Rich in N, O, S Heteroatoms for Sensitive Detection and Efficient Capture of Mercury (II). **2023**. 10.21203/rs.3.rs-3041888/v1.

[5] Y. Ren, Y. Hou, J. Song, D. Zhi, N. Li, Y. Yu and D. Zhu, Rational Design of Regenerable Amino-Functionalized Fluorescent Covalent Organic Framework for the Exclusive Detection of Mercury(II). *Langmuir.,* **2024**, 40, 22990-22996.

[6] Y. J, Xue, A porous thiosemicarbazide functionalized covalent organic framework served as sensitive fluorescent sensor for Hg^{2+}, *J. Food Compos. Anal.,* **2024**, 134, 106500.

[7] N. Gao, X. Chang, Y. Wang, N. Li, W. Guo, Z. Zhao, S. Liu, G. Meng, H. Zhang and B. Wang, A signal amplifying MOF-based probe:on-site and ultrasensitive dual-channel portable detection of Hg^{2+} in groundwater through a fluorimetrically and RGB-based sensing assay. *Talanta.,* **2025**, 286, 127553.
